# Adjustment Disorder in Mental Health Unit of Matosinhos Hospital: a retrospective observational study of 420 patients.

**DOI:** 10.1192/j.eurpsy.2025.1198

**Published:** 2025-08-26

**Authors:** B. Fernández, R. Freitas, T. Valadas, D. Areias

**Affiliations:** 1 Psychiatry, ULSNE, Bragança; 2 Psychiatry, ULSM; 3 Psychiatry, Hospital Magalhaes Lemos, Matosinhos, Portugal

## Abstract

**Introduction:**

Adjustment Disorder (AD), introduced in DSM-III-R, is an emotional or behavioral reaction to a psychosocial stressor that causes significant distress and impairment. Symptoms include excessive worry, sadness, anxiety, and sleep disturbances. U.S. prevalence ranges from 11.5% to 21.5%, higher in those with suicide attempts and twice as common in women. Diagnostic criteria include symptom onset within 1-3 months, significant distress or impairment, and resolution within 6 months after the stressor or its consequences. Subgroups are based on predominant symptoms of anxiety, depression, or behavior (DSM-5).

**Objectives:**

Analysis of Adjustment Disorder cases in patients admitted to medical-surgical Units in 2023 at Hospital de Matosinhos.

**Methods:**

Review of Clinical Records of all internal consultation requests made to the Liaison Psychiatry Team during 2023 at Matosinhos Hospital. Demographic data, psychiatric history, medical-surgical history, multidisciplinary intervention, and post-discharge guidance were obtained.

**Results:**

Out of the 420 patients observed in internal consultation in 2023, 67 were diagnosed with AD, representing 15.9%.

53% were men and 47% women.

The age distribution is represented in figure 1. Most of patients have an age gap between 50-70 years old. 58% is married, 9% single, 9% divorced and 22% widower.

79.1% of the patients live with their family and 20.9% lives alone.

All comorbidities found in patints with adjustment disorder is represented in figure 2. We can see that metabolic diseases and cardiovascular events are the most common comorbidities associated with AD.

Only 32% had seen a psyhiatrist before, while 68% had never contacted with a psychiatrist.

75% of the requests were made by internal medicine and general surgery.

According with the results of figure 3 we can tell that most of the patients started a new medication and just a few had psychological support.

**Image 1:**

**Figure:**
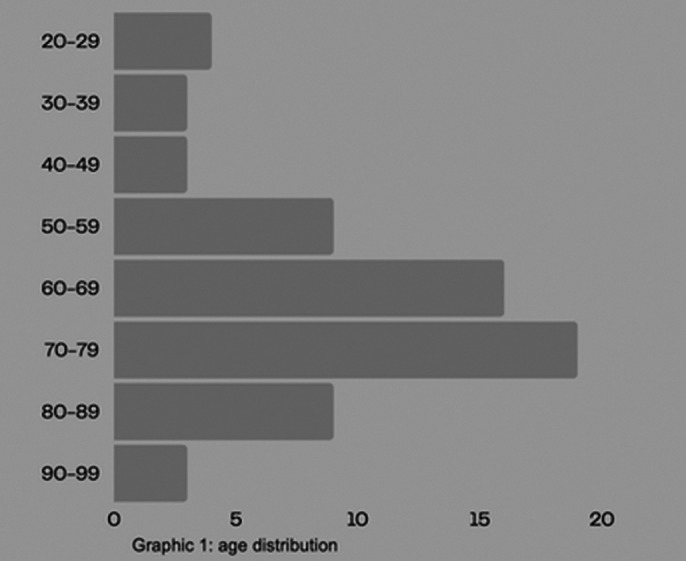

**Image 2:**

**Figure:**
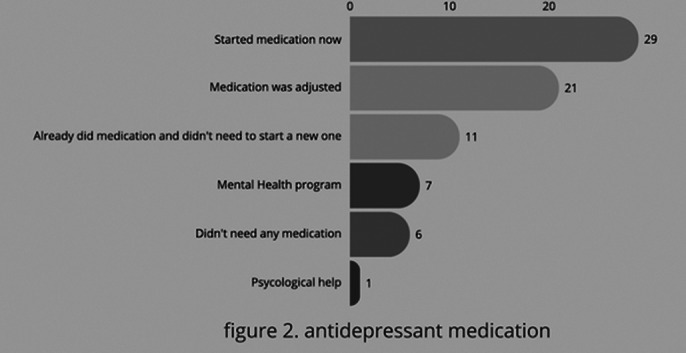

**Image 3:**

**Figure:**
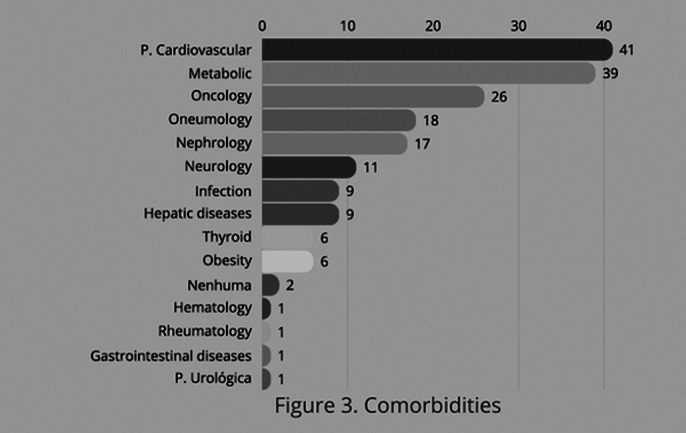

**Conclusions:**

The diagnosis is subject to a degree of subjectivity and requires clinical judgment. There is a lack of clearly defined diagnostic criteria. It depends on a stressor and a reaction to the stressor that is considered excessive relative to what would be expected in the patient’s cultural and social context.

Treatment is underinvestigated. The basic pharmacological management consists of symptomatic treatment of insomnia, anxiety and panic attacks. The use of benzodiazepines to relieve these is common. In this study it is clear the need of giving medication to treat the symptoms even though we know that most of them would stop naturally. However, there is a lack of brief psycological interventions even though they are recommended.

It is now a critical time for advancing our knowledge of the disorder and further studies should be done.

**Disclosure of Interest:**

None Declared

